# Association of novel polymorphisms in *TMEM39A* gene with systemic lupus erythematosus in a Chinese Han population

**DOI:** 10.1186/s12881-017-0405-8

**Published:** 2017-04-20

**Authors:** Xinze Cai, Wenyue Huang, Xudong Liu, Lining Wang, Yi Jiang

**Affiliations:** 1 Central Laboratory, First Affiliated Hospital of China Medical University, Nanjingbei Street, Heping District, Shenyang, 110001 China; 2 Department of Nephrology, First Affiliated Hospital of China Medical University, Nanjingbei Street, Heping District, Shenyang, 110001 China; 3 Central Laboratory; Department of Dermatology, First Affiliated Hospital of China Medical University, Nanjingbei Street, Heping District, Shenyang, 110001 China

**Keywords:** Single nucleotide polymorphism, Systemic lupus erythematosus, Susceptibility

## Abstract

**Background:**

This study aimed to assess the association between 14 single nucleotide polymorphisms (SNPs) in six genes (*IRF8*, *TMEM39A*, *IKZF3*, *ORMDL3*, *GSDMB*, and *ZPBP2*) and systemic lupus erythematosus (SLE) in a Chinese Han population sample.

**Methods:**

We carried out a case-control study of 415 patients with SLE and 470 healthy controls without autoimmune disease or cancer. DNA for genetic analysis was isolated from the blood of all subjects using standard phenol-chloroform method. TagSNPs were identified using genotype data from the panel (Han Chinese in Beijing) of the HapMap Project and were selected using the Haploview program. Genotyping assay was conducted using the Sequenom MassARRAY iPLEX Gold platform. The frequencies of the alleles and genotypes were calculated and analyzed. Association studies and haplotype analysis were also performed.

**Results:**

The genotypic frequencies of rs12493175 and rs13062955 were significantly different between the SLE patients and the healthy controls. Compared with the common homozygous genotype, the CT and CT + TT genotypes in rs12493175 and the AC and AC + AA genotypes in rs13062955 was observed to significantly reduce the risk of SLE. The haplotype analysis of *TMEM39A* polymorphisms showed that the CGTA haplotype frequency was significantly low in the SLE patients.

**Conclusion:**

Our findings identified three novel associations in SNPs located in the *TMEM39A* gene associated with SLE susceptibility in a Chinese Han population.

## Background

Systemic lupus erythematosus (SLE) is typically characterized by the dysregulation of T cell response and B cell activation which usually causes the formation of immune complexes in multiple organs and tissues [1]. Although the pathogenesis of SLE is largely unknown to date, it most likely involves environmental and genetic factors. Several candidate-gene studies and genome-wide association (GWA) scans have successfully discovered multiple susceptibility genes that fall into key pathways implicating immune complex clearance, immune signal transduction and interferon pathways contributing to the development of SLE [2, 3]. However, much of the heritable risk needs to be identified.

Recently, multiethnic approach was utilized to find that three SLE risk loci exceeded the genome-wide significance threshold, including interferon regulatory factor 8 (*IRF8*), transmembrane protein 39A (*TMEM39A*), and 17q21 between IKAROS family of zinc finger 3 (*IKZF3*) and zona pellucida binding protein 2 (*ZPBP2*) [4]. The 17q21 region was originally associated with asthma in family linkage study [5–7]. More single nucleotide polymorphisms (SNPs) in the 17q21 region have been identified as being associated with the susceptibility to autoimmune diseases, including rheumatoid arthritis, ankylosing spondylitis and SLE [4, 8–10]. *IRF8* is a family member of transcription factors that play a critical role in the regulation of cell apoptosis and immune response [11]. It is required for promoting type I interferon responses which can induce the overexpression of genes reported in SLE, and several variants within *IRF8* could influence binding to the regulatory elements [4, 12]. Although very limited biological data of *TMEM39A* is published so far, its polymorphisms have been found to be associated with multiple sclerosis and SLE [4, 13–15]. Additionally, several studies found genetic variants in orosomucoid like 3 (ORMDL3) and gasdermin B (GSDMB) were associated with the risk of autoimmune disease [16, 17]. On the basis of these studies, we hypothesized that certain novel variants in the loci described previously may contribute to the susceptibility to SLE.

In this study, we selected the six candidate genes, namely, *IRF8*, *TMEM39A*, *IKZF3*, *ORMDL3*, *GSDMB*, and *ZPBP2*, and screened for the putatively functional tagSNPs. We aimed to determine the association between the polymorphisms and susceptibility to SLE in a Chinese population.

## Methods

### Sample description

A total of 415 patients with SLE diagnosed according to the criteria of the 1982 American College of Rheumatology were enrolled [18]. Additionally, 470 healthy controls without autoimmune disease or cancer were recruited, who were sex- and age-matched with the patients. All the study participants were from the Chinese Han population, with the age ranging from 16 to 65 years. Demographic and clinical characteristics of SLE patients and controls are shown in Table 1. This project was approved by the Human Ethics Review Committee of China Medical University. Written informed consent was obtained from all the participants, including the guardians on behalf of the children enrolled in the study.

**Table 1:** General characteristics of the study population

	SLE patients (*n* = 415)	Healthy controls (*n* = 470)	*P* value
Female/male	312 (103)	362 (108)	*P* > 0.05
Age (mean ± SD), (years)	38.2 ± 11.4	35.8 ± 13.6	*P* > 0.05
Fever, n (%)	57 (13.7)	-	
Baldness, n (%)	148 (35.7)	-	
Light sensitivity, n (%)	74 (17.8)	-	
Facial erythema, n (%)	161 (38.8)	-	
Oral ulcer, n (%)	67 (16.1)	-	
Arthritis, n (%)	62 (14.9)	-	
Lupus nephritis, n (%)	246 (59.3)	-	

### SNP selection

TagSNPs are representative SNPs which can capture most of the genetic variation in a region of the genome on the basis that they are in high linkage disequilibrium (LD) with other SNPs [19]. TagSNPs genotyped in this study were selected by analyzing the genotype data of Chinese Han population from HapMap dbSNP (http://www.hapmap.ncbi.nlm.nih.gov) using LD-based tagSNP selection with a pairwise algorithm LDSelect, available in the Tagger function implemented in Haploview version 4.2 (http://www.broadinstitute.org/mpg/haploview) [20, 21].

First, genotype data of HapMap Chinese Han Beijing population (Release 27, Phase I + II + III) were extracted and the chromosomal regions including the six candidate genes within the extended gene regions encompassing 3000 bp upstream and 1500 bp downstream flanking sequence (to capture the 5’ and 3’ UTR) were searched. TagSNPs were chosen based on a minor allele frequency (MAF) of at least 5% and a pairwise LD threshold of *r* ^2^ > 0.8 using Haploview 4.2. Second, the F-SNP program (http://compbio.cs.queensu.ca/F-SNP) and SNP Function Prediction (FuncPred) software (http://snpinfo.niehs.nih.gov/snpinfo/snpfunc.htm) were applied to prioritize the tagSNPs for genotyping based on their putative functions. Accordingly, 14 tagSNPs with predicted functional effects were selected for genotyping. The common SNPs captured using the selected tagSNPs in the six candidate genes are presented in Table 2.

**Table 2:** Common SNPs captured using the selected 14 tagSNPs in the six candidate genes based on the HapMap population data for Chinese in Beijing (Release 27)

Gene	tagSNP_ID	SNP captured	Position (hg19)
*IKZF3*	rs3816470	rs9635726, rs3816470, rs9303277, rs10445308, rs9909593	chr17:37985801
	rs907092	rs907092	chr17:37922259
*GSDMB*	rs9303281	rs11078927, rs1008723, rs4795400, rs9303281, rs2305480, rs7219923, rs869402, rs2305479, rs7224129, rs11078926, rs2290400, rs7216389	chr17:38074046
*ORMDL3*	rs4795402	rs4795403, rs4795402, rs3744246, rs4795404	chr17:38085385
	rs8076131	rs4378650, rs8076131, rs12603332	chr17:38080912
*ZPBP2*	rs11557466	rs11557467, rs12936231, rs11078925, rs1054609, rs11557466, rs11870965, rs10852936, rs9907088	chr17:38024626
*IRF8*	rs188602	rs188602, rs170033, rs2270502, rs381139	chr16:85932351
	rs4843860	rs4843860, rs12926854, rs4843861	chr16:85950921
	rs2270501	rs2270501, rs12924316	chr16:85932988
	rs191022	rs191022	chr16:85932132
*TMEM39A*	rs13062955, rs12493175	rs12492859, rs13094625, rs13081197, rs13078312, rs12493326, rs16829853, rs13081067, rs2282170, rs13062955, rs12492315, rs12493175, rs13096213, rs12496277, rs12492609	chr3:119159658,chr3:119160413
	rs4687859	rs7629750, rs2282171, rs3772136, rs9846088, rs4687859, rs9872589, rs3195852	chr3:119170371
	rs2282175	rs17281647, rs2282175, rs1132202	chr3:119182259

### Genotyping assay

Genomic DNA was isolated from peripheral blood leukocytes using the standard phenol-chloroform method. Each DNA sample was diluted to working concentration of 50 ng/μl for genotyping. The selected tagSNP genotyping was performed by BGI (Shenzhen, China) using the Sequenom MassARRAY iPLEX Gold platform (Sequenom, San Diego, California) according to the manufacturer’s instructions [22]. The primers for polymorphism genotyping were designed using MassARRAY Assay Design 3.1 software and are shown in Table 3. All samples were randomized on 384-well plates and blinded for case or control status. A random selection of samples was repeatedly genotyped using direct sequencing validate the accuracy of the SNP genotyping assays and the results were 100% concordant.

**Table 3:** Details of the primers used in the polymorphism genotyping by MassArray

tagSNP_ID	Alleles	Forward and reverse primer	Extension primer
rs2282175	C/T	ACGTTGGATGGAAAGCGGCGACAACTTTAC	CGCTGGGAGGGAGTTC
		ACGTTGGATGCTGGTTTGCAGCGTTCCAAC	
rs4687859	A/G	ACGTTGGATGCATGCCTGGCCTCATTTTTC	TTTTCCCTGCCTCATTG
		ACGTTGGATGAGAAAGCACATTTCCCTGCC	
rs12493175	C/T	ACGTTGGATGGTTATGGGACAGCTTCTTTC	CCCAAACGTATGAAGGTTAACAG
		ACGTTGGATGGAGAGGTGAGAAAGCTACAG	
rs13062955	A/C	ACGTTGGATGGGCAAATACAGGCATACCTC	GGACTACAGTATCTGGGAAGCACAAT
		ACGTTGGATGGGGTTGCCACAAACCTTCAG	
rs9303281	A/G	ACGTTGGATGACCCCTTTTTTGGACTCAGC	CTCTTCCATGTGAAGAGAGTCCA
		ACGTTGGATGACGTGCGTCCATGTGAAGAG	

### Reverse transcriptase–PCR of candidate gene mRNA levels

To examine the relation between the associated polymorphisms and the gene mRNA levels, forty patients stratified by polymorphic genotypes were randomly selected. The relative expression levels of twenty patients with common homogenous genotype carriers were set to a unity, and the relative expression levels of twenty patients with heterogeneous and rare homogenous genotypes were expressed relative to those of the common homogenous genotype carriers. Total RNA prepared from peripheral blood mononuclear cells were reversely transcribed using the TaqMan reverse transcription reagents (Applied Biosystems, Weiterstadt, Germany). RT-PCR was carried out using the ABI Universal Master Mix on an ABI PRISM 7000 Sequence Detection System.

### Statistical analysis

Data were managed and stored using the SPSS software 16.0. Allele and genotype frequencies were compared between patient and control groups by the chi-square (*χ* ^2^) test. The quality of the genotype data was evaluated by Hardy-Weinberg equilibrium (HWE) in the case and control subjects using Fisher’s exact test (*P* > 0.05). The association between each polymorphism and risk of SLE was estimated by logistic regression and was expressed as odds ratio (OR) with 95% confidence intervals (95% CI). The haplotypes were assigned using the online software platform SHEsis (http://www.analysis.bio-x.cn). The haplotype construction element is based on the standard Full-Precise-Iteration (FPI) algorithm [23]. All tests were two-tailed, and *P* values < 0.05 were considered as statistically significant.

## Results

In the screening stage, a few of tagSNPs for the six candidate genes were excluded from further analysis because they were found to have no polymorphic sites or to exhibited MAFs < 0.05 in Chinese Han Beijing population. Finally, 14 tagSNPs with predicted functional effects were selected for genotyping in a total of 885 subjects (Table 2). The details of the five identified genetic single-nucleotide variants in two genes, namely, *TMEM39A* rs2282175, rs4687859, rs12493175, rs13062955, and *GSDMB* rs9303281 were presented (Table 4). Additionally, there was no significant difference concerning the call rates between the SLE group and the control (*p* > 0.05). However, as shown in Table 5, the allelic distributions of rs4687859 and rs9303281 showed significant departure from the Hardy-Weinberg law for the controls. The allelic distributions of the three selected tag-SNPs, rs2282175, rs12493175, and rs13062955, of the *TMEM39A* gene met the Hardy-Weinberg principle (Table 5). Thus, we focused on the three selected tag-SNPs, rs2282175, rs12493175, and rs13062955, of the *TMEM39A* gene in the following analysis (Table 6).

**Table 4:** The details of the identified genetic single-nucleotide variants

SNP	Chr	Position	Func.refGene	CADD.Score
rs13062955	chr3	119159658	intronic	CADD = 3.564
rs12493175	chr3	119160413	intronic	CADD = 2.429
rs4687859	chr3	119170371	intronic	CADD = 8.630
rs2282175	chr3	119182259	UTR5	CADD = 4.988
rs9303281	chr17	38074046	intronic	CADD = 0.720

*Func.refGene* functional gene element, *CADD* Combined Annotation Dependent Depletion

**Table 5:** The five SNPs call rates in patients and control individuals and HWE *p*-values

SNP_ID	Call rate (%)		HWE *p*-value	
	SLE	Control	SLE	Control
rs2282175	98	99	0.539349	0.056740
rs4687859	98	95	0.792124	0.000719
rs12493175	99	99	0.004372	0.295414
rs13062955	97	92	0.002761	0.107666
rs9303281	93	99	7.77E-16	2.66E-15

**Table 6:** Genotype and allele association analysis of three tagSNPs

tagSNP_ID	Genotype/Allele	SLE, n(%)	CON, n(%)	*χ* ^2^	*P* value	OR (95% CI)	*P* value	P.adjust
rs2282175	CC	339 (83.7)	415 (88.9)	5.1	0.08	1		
	CT	62 (15.3)	48 (10.3)			1.63 (1.06–2.38)	0.026	0.054
	TT	4 (1.0)	4 (0.9)			1.21 (0.30–5.00)	0.776	0.817
	CT/TT					1.56 (1.05–2.27)	0.027	0.054
	C	740 (91.4)	878 (94.0)	4.5	0.033	1		
	T	70 (8.6)	56 (6.0)			1.49 (1.03–2.12)	0.033	0.06
rs12493175	CC	311 (75.7)	308 (66.2)	12.7	0.002	1		
	CT	85 (20.7)	145 (31.2)			0.58 (0.42–0.79)	0.001	0.005
	TT	15 (3.6)	12 (2.6)			1.23 (0.57–2.70)	0.589	0.736
	CT/TT					0.62 (0.46–0.84)	0.002	0.007
	C	707 (86.0)	761 (81.8)	5.6	0.017	1		
	T	115 (14.0)	169 (18.2)			0.73 (0.56–0.95)	0.017	0.0486
rs13062955	CC	305 (75.9)	281 (66.0)	15.1	0.001	1		
	AC	82 (20.4)	136 (31.9)			0.55 (0.40–0.76)	2.95 × 10^−4^	0.002
	AA	15 (3.7)	9 (2.1)			1.53 (0.66–3.57)	0.318	0.424
	AC/AA					0.61 (0.45–0.84)	0.002	0.007
	C	692 (86.1)	698 (81.9)	5.2	0.021	1		
	A	112 (13.9)	154 (18.1)			0.73 (0.56–0.96)	0.021	0.053

P.adjust: the Bonferroni corrected *P* value

The genotypic frequencies of rs12493175 and rs13062955 located in *TMEM39A* gene were significantly different between the SLE patients and the healthy controls. Compared with the common homozygous genotype, the CT and CT + TT genotypes in rs12493175 (p.adjust = 0.005, odds ratio (OR) 0.58, 95% CI 0.42 to 0.79; p.adjust = 0.007, OR 0.62, 95% CI 0.46 to 0.84, respectively) and the AC and AC + AA genotypes in rs13062955 (p.adjust = 0.002, OR 0.55, 95% CI 0.40 to 0.76; p.adjust = 0.007, OR 0.61, 95% CI 0.45 to 0.84, respectively) was observed to significantly reduce the risk of SLE. On the other hand, the difference in the frequency of rs2282175 was only marginal. The CT and CT + TT genotypes in rs2282175 was observed to modestly increase the risk of SLE (p.adjust = 0.054, OR 1.63, 95% CI 1.06 to 2.38; p.adjust = 0.054, OR 1.56, 95% CI 1.05 to 2.27, respectively). However, we did not find any other tagSNP associated with SLE risk in the genes of *IRF8*, *IKZF3*, *ORMDL3*, *GSDMB* and *ZPBP2*. We also evaluated the relation between the associated polymorphisms and the gene mRNA levels in peripheral blood mononuclear cells from 40 patients. Nevertheless, we failed to find any correlation between them (data not shown).

Haplotypes were constructed in both SLE and healthy controls and the haplotypes with frequency of > 3% were built from *TMEM39A* rs2282175, rs12493175 and rs13062955 (Table 7). The results show that the CGTA haplotype frequency was significantly low in the SLE patients (*p* = 0.019, OR 0.72, 95% CI 0.55 to 0.95). No difference was detected in the other haplotypes.

**Table 7:** Frequencies of the haplotypes formed by *TMEM39A* rs2282175, rs12493175 and rs13062955 SNPs

Haplotype	SLE, n(%)	CON, n(%)	*P* value	OR (95% CI)
CACC	492.8 (62.4)	527.7 (63.0)	0.813	0.97 (0.79–1.19)
CGCC	119.4 (15.1)	103.3 (12.3)	0.101	1.26 (0.95–1.68)
CGTA	108.7 (13.8)	151.0 (18.0)	0.019	0.72 (0.55–0.95)
TGCC	65.5 (8.3)	52.7 (6.3)	0.11	1.34 (0.92–1.96)

## Discussion

*IRF8*, *TMEM39A* and *IKZF3*-*ZPBP2* were previously identified as susceptibility loci for SLE in the multiracial replication study [4], Besides, *ORMDL3* and *GSDMB* were found to have susceptibility loci for autoimmune diseases [16, 17]. Thus we hypothesized certain novel associations in SNPs located in these genes could be identified in Chinese populations. To test this hypothesis, we selected 14 tagSNPs in these candidate genes to determine the association between the polymorphisms and SLE susceptibility in a Chinese Han population. Our findings showed that *TMEM39A* rs2282175, rs12493175, and rs13062955 were associated with SLE risk.

To date, almost no biological data of *TMEM39A* have been reported and only two SNPs in *TMEM39A* were identified as being associated with the susceptibility of autoimmune diseases. *TMEM39A* rs1132200 have been found to be associated with susceptibilities to multiple sclerosis and SLE in multiracial replication study [4, 13, 14]. but the recent studies showed that *TMEM39A* rs12494314, instead of rs1132200, was associated with SLE susceptibility in the Chinese population [15, 24]. In our current study, we identified three novel associations in SNPs located in *TMEM39A* as being associated with SLE susceptibility. The genotypic frequencies of rs12493175 and rs13062955 were significantly different between the SLE patients and the healthy controls, while the difference in the frequency of rs2282175 was only marginal. Among these polymorphisms, rs12493175 T-allele and rs13062955 A-allele were found to be associated with a reduced SLE risk, suggesting a protective factor to SLE. In contrast, rs2282175 T-allele was found to be associated with an increased SLE risk, suggesting a susceptibility factor to SLE. Haplotype analysis for *TMEM39A* SNPs revealed that the haplotype CGTA conferred a reduced risk of SLE. It is possible that the haplotype CGTA provides protection to SLE, resulting from the rs12493175 T and rs13062955 A alleles. It is worth noting that rs2282175 is located in the region of 5' upstream in *TMEM39A* and predicted to be a binding site of certain transcription factor. It is speculated that the C → T allele change at the rs2282175 site may influence the DNA binding ability of transcription factor c-Rel, which was predicted according to the different variants using the search tool of Alibaba 2.1 (http://www.gene-regulation.com/pub/programs/alibaba2). Although we did not find any relation between mRNA expression and the polymorphisms, it may be required to explore the possible biological significance of the SNPs in different cell subsets.

Several limitations in the current study should also be noted. First, due to the restricted number of study subjects and limited analysis capacity, we did not analyze the SNPs with rare MAF in the Chinese Han population, including those reported as risk loci for SLE in other ancestries, and mainly focused on the SNPs with predicted functional effect. Further research on the role of the rare variants should be carried out in a larger number of samples. As different populations have different genetic backgrounds, it is still necessary to perform the genetic analysis of multiracial study. Second, we still could not determine the causality of SLE-associated SNPs. For those variants in the large strong LD region, such as chromosome 17q21, it is difficult to determine which SNP is the true functional locus that contributes to SLE susceptibility independently. Better understanding whether the SNP is functionally relevant will require mechanistic and fine-mapping experiments. Third, our study did not assess the SNP-SNP interaction (epistasis). For the genetically complex disease, multiple interacting loci could contribute to SLE susceptibility. Additionally, as a heterogenetic disease, the contribution of genetic and environmental factors is very important for the disease [25]. Therefore, interaction between genetic and environmental factors is required to further clarify the pathogenesis of SLE.

## Conclusion

This study identified three novel associations in SNPs located in the *TMEM39A* gene associated with SLE susceptibility in a Chinese Han population. Functional study and further independent large-scale study in other racial populations are still needed to confirm our results.

## Abbreviations

CI: Confidence interval
GSDMB: Gasdermin B
GWA: Genome-wide association
IKZF3: IKAROS family of zinc finger 3
IRF8: Interferon regulatory factor 8
MAF: Minor allele frequency
OR: Odds ratio
ORMDL3: Orosomucoid like 3
SLE: Systemic lupus erythematosus
SNP: Single nucleotide polymorphism
TMEM39A: Transmembrane protein 39A
ZPBP2: Zona pellucida binding protein 2
